# Nine quick tips for trustworthy machine learning in the biomedical sciences

**DOI:** 10.1371/journal.pcbi.1013624

**Published:** 2025-10-30

**Authors:** Luca Oneto, Davide Chicco

**Affiliations:** 1 Dipartimento di Informatica Bioingegneria Robotica e Ingegneria dei Sistemi, Università di Genova, Genoa, Italy; 2 Dipartimento di Informatica Sistemistica e Comunicazione, Università di Milano-Bicocca, Milan, Italy; 3 Institute of Health Policy Management and Evaluation, University of Toronto, Toronto, Ontario, Canada; SIB Swiss Institute of Bioinformatics, SWITZERLAND

## Abstract

As machine learning (ML) becomes increasingly central to biomedical research, the need for trustworthy models is more pressing than ever. In this paper, we present nine concise and actionable tips to help researchers build ML systems that are technically sound but ethically responsible, and contextually appropriate for biomedical applications. These tips address the multifaceted nature of trustworthiness, emphasizing the importance of considering all potential consequences, recognizing the limitations of current methods, taking into account the needs of all involved stakeholders, and following open science practices. We discuss technical, ethical, and domain-specific challenges, offering guidance on how to define trustworthiness and how to mitigate sources of untrustworthiness. By embedding trustworthiness into every stage of the ML pipeline – from research design to deployment – these recommendations aim to support both novice and experienced practitioners in creating ML systems that can be relied upon in biomedical science.

## Introduction

The current generation of Artificial Intelligence (AI) tools in the biomedical sciences is predominantly based on inductive AI, specifically machine learning (ML) [1], which leverages data to generate new knowledge [2]. Applications range from the use of large language models for clinical diagnosis [3] to addressing the protein folding problem with advanced deep learning architectures [4]. In contrast, deductive AI [5] operates by reasoning over well-established knowledge, for example, to plan hospital procedures [6] or define clinical pathways [7]. As ML discovers patterns by analyzing data and identifying correlations [8], its trustworthiness can be limited [9], and its performance is highly dependent on the quality and quantity of the input data [10]. The widespread adoption of AI, along with the resulting surge in data usage and rising societal concerns about its untrustworthiness, are among the main reasons behind the development of regulations such as the Data-Act [11] and the AI-Act [12] in the European Union. Beyond the obvious risks of intentional misuse, the current generation of AI-based systems, being highly untrustworthy, may lead to unintentional misuse [13]. Nevertheless, striking an appropriate balance between the need for regulation and the advancement of biomedical sciences remains a significant challenge [14].

Trustworthiness is the property of an ML-based system that emerges from the integration of technical robustness [15], ethical responsibility [16], and domain awareness [17], ensuring that its behavior is reliable, transparent, and contextually appropriate for biomedical applications. From a technical standpoint [15], ML-based system can accumulate technical debt, such as fragile data pipelines, poorly versioned models, and lack of robust monitoring, that makes them difficult to maintain and prone to failure, particularly in operational environments. Ethically [16], ML-based system may violate principles of privacy through excessive data collection or poor anonymization, exhibit bias and lack fairness due to skewed training data, and lack robustness and explainability, making them unreliable in unpredictable settings and opaque to end-users and stakeholders. Lacks of domain knowledge [17] result in ML-based system that might capture statistical correlations but miss clinically meaningful insights, potentially leading to unsafe or ineffective recommendations. In fact, if contextual information is not incorporated to appropriately support the collected data, we might identify non-causal relationships that could lead to the model failing to generalize or, even more concerning, producing unsafe or ineffective recommendations.

To address the technical, ethical, and domain-specific challenges of deploying ML-based systems, we propose a series of quick tips for fostering trustworthy ML in the biomedical sciences. These concise and practical recommendations are not intended to be exhaustive; rather, they aim to raise awareness among novices while remaining equally valuable for experienced practitioners.

## Tip 1: Think about the consequences of your actions

Our advice is straightforward: before applying an ML method to biomedical data, carefully evaluate all potential consequences, with particular attention to possible negative outcomes. This advice is intended for all machine learning researchers and to anyone reading this article and planning to perform a machine learning analysis of biomedical data. Scientific projects, despite their intentions, can lead to unintended consequences, some of which may cause significant harm.

Examples of such unintended negative outcomes include adverse drug reactions and misinterpretations of data. For instance, drugs discovered through computational methods may present unforeseen side effects not evident during clinical trials, potentially leading to health problems for patients [18]. Likewise, findings from medical informatics studies may be misinterpreted or overgeneralized, resulting in inappropriate clinical practices or misguided public health policies [19].

Some biomedical informatics studies may be problematic not because of their findings, but due to their origins. A well-known example is the thousands of studies conducted using HeLa cells, derived from the tissue of Henrietta Lacks without her consent [20].

To mitigate such risks, we recommend considering all stakeholders involved in a study (such as ML researchers, biomedical scientists, clinicians, study designers, patients, grant holders, and legal or administrative personnel) and carefully reflecting on the potential consequences—positive and negative, intended and unintended—of the research outcomes. The boundary between good and bad is often ambiguous. Even outcomes that violate an individual’s rights may be tolerated when framed as low-risk AI use or as serving the greater good.

The AMIA 2009 Health Policy Meeting [21] proposed a useful framework for categorizing consequences: anticipated (direct or indirect) and unanticipated (direct or indirect). Each of these categories can be further classified as desirable or undesirable. Additionally, consequences may be temporally segmented into short-term (up to six months), medium-term (six months to three years), and long-term (beyond three years). Again, however, the distinction between short-term and long-term is fuzzy. Some consequences might come with undesirable short-term change problems which can also affect patients negatively soon, but ultimately in long run benefits more people. Further insights into the unintended consequences of biomedical information technologies can be found in the specialized literature [22,23].

## Tip 2: Define the concept of trustworthiness for your specific biomedical applications

The meaning of trustworthiness can vary depending on the domain, data, and other contextual factors. Importantly, trustworthiness is also dynamic: what is deemed trustworthy may shift over time as technologies evolve, regulatory frameworks change, or societal expectations develop.

For example, Cutillo and colleagues [24] define trustworthiness as “the ability to assess the validity and reliability of a machine intelligence-derived output across varying inputs and environments.” Park and colleagues [25] associate trustworthiness with providing users interpretable information that enhances their confidence in diagnostic results and potentially influences their decisions. Similarly, Huang and colleagues [26] describe trustworthiness as the provision of reliable and understandable information extracted from magnetic resonance images, thereby supporting diagnostic differentiation for medical doctors.

Given these differing perspectives, we caution against adopting a single, rigid definition of trustworthiness. Instead, researchers should explore and adapt context-appropriate notions of trustworthiness to their specific scientific setting. Two main strategies can be pursued. First, biomedical informatics may be divided into three principal subfields (bioinformatics, health informatics, and chemoinformatics [27]) and the role of trustworthiness within each. Alternatively, one could classify the field according to data types—such as genomics, proteomics, transcriptomics, medical images, electronic health records, drug compounds, and biosignals—and analyze what “trustworthy” means in the context of each category.

In either case, the analysis should begin by identifying the actors involved with the ML-based tool (Tip 4). Subsequently, the three primary dimensions of trustworthiness should be considered: technical (Tip 5), ethical (Tip 6), and domain-specific (Tip 7). Clarifying trustworthiness in this way directly shapes system design, evaluation criteria, and ultimately the likelihood of stakeholder adoption [28–31].

It is important to notice that the meaning and definition of trustworthiness also differ from the perspective of each actor (Tip 4).

## Tip 3: Be aware of what can and what cannot be done to achieve trustworthiness

This tip defines theoretical boundaries of trustworthiness metrics. Trustworthiness in biomedical ML can be pursued through two conceptually distinct routes, each with its own strengths and limitations [32].

The first route involves embedding trustworthiness directly into the learning objective, instructing the model itself to discover and exhibit trustworthy behavior [33], for example, through approaches such as neuro-symbolic ML [34]. Although conceptually appealing, this strategy remains largely aspirational: it faces unresolved philosophical questions and the practical challenge of translating abstract notions of trustworthiness into precise mathematical formalisms.

The second route takes a pragmatic approach: trustworthiness is operationalized through a portfolio of carefully human-defined quantitative metrics [16]. These include ethical metrics: fairness [35], including demographic parity or counterfactual fairness; explainability [36], encompassing intrinsic or post-hoc approaches, model-specific or model-agnostic methods, and global or local perspectives, whether feature- or example-based; robustness [37], with respect to both natural and adversarial perturbations; and privacy guarantees [38], ranging from mitigation strategies based on differential privacy to stronger protections relying on cryptographic protocols. Additional technical dimensions [39] involve compliance with regulations, validity, accountability, replicability, and computational or energy requirements. Finally, domain-specific considerations [40] address clinical validity and utility, alignment with biomedical knowledge, conformity with medical standards and regulations, and the integration of expert feedback.

This second strategy has achieved remarkable results, yet it is not without limitations. First, most metrics are aggregated, capturing only average behavior. Pointwise guarantees, which would ensure safe performance for every individual patient or rare yet clinically critical sub-populations, generally require symbolic reasoning and remain elusive [41]. Second, many desiderata are mutually incompatible, as illustrated by fairness impossibility results [42] and tensions between competing trustworthiness metrics [16]. Certain dimensions of trustworthiness are actually binary. For instance, when strict confidentiality is required [38], differential privacy or anonymization can only mitigate risk. Stronger guarantees demand cryptographic protocols such as secure multi-party computation or homomorphic encryption, which severely restrict the set of feasible algorithms and inflate computational costs. Some metrics are also ill-posed. Explainability exemplifies this challenge: post-hoc techniques provide useful probes of decision surfaces but only approximate, rather than faithfully reveal, the internal causal logic of opaque models [36]. Unless interpretability is built in by design—through sparse rule sets or symbolic programs—explanations should be presented as hypotheses, not ground truths, and validated through user studies or counterfactual tests. Finally, optimizing any single dimension of trustworthiness can expose hidden vulnerabilities elsewhere, triggering cascades of additional diagnostics [43]. Iterative, multi-faceted evaluation, therefore, becomes a continuous obligation rather than a one-time certification step [44].

Nevertheless, while human oversight should be embedded in ML-based systems, human decisions still rely on the quality of the underlying technical components; relying solely on qualitative, human-based judgments can be equally risky. Striking the right balance is crucial—human-defined criteria may take the lead, but technical rigor remains essential.

## Tip 4: Look for a compromise between all involved parties

Biomedical informatics projects often involve multiple stakeholders, including ML researchers, biomedical scientists, clinicians, study designers, patients, grant holders, and legal or administrative personnel. These roles bring distinct priorities and interpretations of trustworthiness. For example, patients may prioritize data privacy [45], mental health professionals may emphasize transparency and explainability [46], while researchers and editors may focus on methodological rigor and adherence to publication standards [47].

We recommend that these perspectives be carefully balanced, with patients placed at the top of the priority list [48, Rule 4]. Not all constraints are negotiable: legal and ethical frameworks—such as Data- and AI-act—may impose strict limitations, while budgetary or methodological restrictions can preclude otherwise desirable analyses. Compromise must therefore extend beyond scientific objectives to encompass practical implementation, communication strategies, and publication venues. To make this process actionable, concrete engagement techniques such as stakeholder interviews, co-design workshops, consensus-building approaches, or Delphi panels can be employed to elicit and integrate diverse perspectives [49]. Open and inclusive discussion remains essential, with the recognition that perfect consensus is rare and some dissatisfaction is inevitable [50]. To avoid analysis paralysis, a transparent decision-making process should be established, ensuring that trade-offs are openly documented and justified.

## Tip 5: Take care of the technical aspects

This tip provides technical tools to address Tip 3’s limits. Biomedical datasets are outpacing Moore’s law, fueled by high-resolution imaging, next-generation sequencing, and continuous remote monitoring [51]. Harnessing hardware accelerators and distributed frameworks enables laboratories to iterate on large models while keeping training times practical [52,53]. Yet raw speed is only one side of the coin: computation, energy, and memory footprints also matter [54]. Techniques such as mixed-precision training, quantization, pruning, and knowledge distillation can shrink model size without sacrificing diagnostic accuracy [55].

Prototype notebooks often graduate directly into production dashboards, accumulating hidden technical debt that hampers future innovation [39]. Adopting modular, version-controlled pipelines, augmented with automated unit and integration tests and infrastructure-as-code templates, helps systems remain malleable while satisfying good machine-learning-practice guidelines [56].

Biomedical ML must satisfy both domain-agnostic and domain-specific regulation [11,12,57]. Core practices include strict data-provenance logging, privacy-preserving pipelines, and routine model-governance audits that trace every prediction back to a versioned model artifact and dataset snapshot [56,58,59]. This is tightly connected with the concept of accountability [60,61] requiring continuous monitoring, standard governance artifact, sign-off from domain experts, data stewards, and compliance officer.

Classic benchmarks seldom capture the nuances of real-world clinical deployment [62]. In addition to accuracy, teams should track diverse metrics and log every experimental choice to facilitate audits and exact reproduction during publication or regulatory review [63,64]. The rise of billion-parameter foundation models introduces further operational burdens: teams must track the provenance of web-scale training corpora, expunge sensitive content, and apply safe, domain-specific fine-tuning [56]. Table 1 presents typical scopes along with the associated tasks and libraries that handle the technical aspects.

**Table 1:** Typical scopes along with the associated tasks and libraries that handle the technical aspects.

Scope	Tasks	Libraries
Hardware accelerators and distributed training	GPU/TPU utilization, multi-node scaling, memory sharding	CUDA/cuDNN, ROCm, oneAPI, PyTorch DDP/FSDP, DeepSpeed (ZeRO), Horovod, Ray Train, Lightning Fabric, Hugging Face Accelerate, Megatron-LM, JAX + XLA
Efficient training (mixed precision, quantization)	Automatic mixed precision, post-training, or quantization-aware training	PyTorch AMP, NVIDIA Apex, JAX bf16, TF mixed precision, ONNX Runtime Quantization, TensorRT INT8, OpenVINO, Intel Neural Compressor, bitsandbytes (8/4-bit)
Efficient inference and serving	Low-latency serving, graph optimization	TensorRT, Triton Inference Server, TorchTensorRT, TF-TRT, ONNX Runtime, vLLM, Text Generation Inference (TGI), TorchServe, TF Serving, BentoML, KServe
Pruning and sparsity	Structured/unstructured pruning, sparse kernels	PyTorch pruning, TensorFlow Model Optimization Toolkit (TF-MOT), SparseML/DeepSparse, NNI pruning
Parameter-efficient fine-tuning	LoRA/QLoRA, adapters	PEFT (HF), LoRA, QLoRA, Adapters (adapter-transformers)
Knowledge distillation	Teacher-student training	torchdistill, Hugging Face Transformers distillation scripts, TextBrewer (NLP), TF-MOT distillation
Notebook to maintainable pipeline	Pipelines, orchestration, modularity	Dagster, Prefect, Airflow, Kubeflow Pipelines, Metaflow, Luigi
Version control (code and data)	Git, data artifacts, datasets	Git/Git LFS, DVC, lakeFS, Pachyderm, Quilt, Delta Lake/Iceberg/Hudi
Testing (unit/integration/data)	CI, data quality, ML-specific tests	pytest, hypothesis, Great Expectations, Soda, DeeChecks, Giskard
Infrastructure as code and environments	Reproducible infrastructure, kubernetes, packaging	Terraform, Pulumi, CloudFormation, Helm/Kustomize, Docker/Podman, Poetry/Conda
Experiment tracking and reproducibility	Runs, params, artifacts, registries	MLflow (Tracking + Model Registry), Weights and Biases, Neptune, Comet, Sacred
Data provenance and lineage	Track sources, transformations	OpenLineage/Marquez, DataHub, OpenMetadata, Amundsen, MLMD
Governance artifacts	Model/dataset cards, documentation	Model Card Toolkit, Responsible AI Dashboard (Azure), Fairlearn, AIF360
Security and confidential compute	Encrypted/isolated training and inferencing	OpenFHE/SEAL/PALISADE (HE), SGX/TDX runtimes (e.g., Gramine), HashiCorp Vault, OPA
Compliance workflow and audits	Evidence collection, approvals, trials	Jira/ServiceNow integrations, Confluence, Audit log exporters, Schellman Evidence, Vanta (general GRC)
Monitoring and drift	Data/label drift, performance, alerts	Evidently, whylogs/WhyLabs, Arize AI, Fiddler, Seldon Alibi Detect, SageMaker Model Monitor, Vertex Model Monitoring
Benchmarking and eval (beyond accuracy)	Robustness, fairness, clinical realism	MedPerf (medical eval), RobustBench, HoloClean (data), Fairlearn/AIF360, HELICON/evals (LLM evals), Promptfoo
Foundation-model data curation	Corpus filtering, dedup, licensing	ccnet, datatrove (HF), Dolma (AI2), Cleanlab, datasketch (MinHash), scancode-toolkit, reuse-tool
Safety and content filtering	PII/toxicity/medical-risk filters	Presidio, Detoxify, Perspective API (service), OpenAI Safety Spec tooling analogs, LAION NSFW detectors
Deployment in clinical settings	API/serving, rollouts, A/B testing, canary releases	BentoML, KServe, Seldon Core, Istio (canary), FastAPI

## Tip 6: Take care of the ethical aspects

This tip offers ethical tools to complement Tip 3’s boundaries. Systematic biases in electronic health records, biobanks, or imaging repositories can propagate directly into clinical-decision tools, widening existing health disparities [35,65]. Before model development, audit cohort composition with respect to sensitive attributes and simulate counterfactual performance gaps using a-priori fairness metrics. If substantial imbalance is detected, apply pre-, in-, or post-processing mitigation strategies.

Regulators increasingly expect human-interpretable rationales for ML-driven recommendations in patient care [36,66]. Use explainable models by design where feasible, or apply post-hoc methods when interpretability must be added after training. Combine model-specific tools (for example, gradient-based) with model-agnostic ones (for example, perturbation-based) to strengthen trust. Global explanations should confirm alignment with biomedical mechanisms, while local explanations—whether feature-based or example-based—should provide per-patient justifications that clinicians can assess. Natural and adversarial perturbations to medical images can flip a cancer diagnosis, and data-poisoning attacks can smuggle malicious correlations into federated genomic models [37]. Before clinical validation, subject the entire inference stack to testing, formal verification for worst-case guarantees on high-risk thresholds, and scenario-based simulations that probe rare but plausible edge cases [67].

Strict data-protection regimes restrict the handling of personal biomedical data [38]. Differential privacy and secure multiparty computation have matured to the point where they can be embedded into everyday ML pipelines. If data sovereignty laws prevent centralization, consider federated or split-learning protocols backed by secure multiparty computation or homomorphic encryption. Table 2 presents typical scopes along with the associated tasks and libraries that handle the ethical aspects.

**Table 2:** Typical scopes along with the associated tasks and libraries that handle the ethical aspects.

Scope	Tasks	Libraries
Bias and fairness auditing	Audit cohort composition, simulate counterfactuals, compute fairness metrics	AIF360 (IBM), Fairlearn, Themis-ML, EthicalML/Fairness Indicators, aequitas, Giskard
Bias mitigation (pre/in/post)	Rebalancing, adversarial debiasing, reweighting, equalized odds	AIF360, Fairlearn, TensorFlow Constrained Optimization, FairTorch
Explainability (global and local)	Post-hoc explanations, interpretable models	SHAP, LIME, Captum (PyTorch), ELI5, InterpretML, inherently interpretable models: Explainable Boosting Machine, GLRM
Biomedical-specific explainability	Align explanations with biomedical knowledge, feature attributions	BioSHAP (biomedical SHAP adaptations), Klarity AI (radiology), Integrated Gradients (Captum/JAX), Grad-CAM/Score-CAM for imaging
Robustness and adversarial testing	Image perturbations, poisoning simulations, adversarial robustness	CleverHans, Adversarial Robustness Toolbox (ART), Foolbox, TextAttack (for NLP), DeepRobust
Formal verification and safety checks	Provable guarantees, threshold certification	ERAN (ETH Robustness Analyzer), Marabou, AI2’s Reluplex, DeepCert, DeepPoly
Scenario-based simulations	Rare/edge-case probing, stress testing	Carla (causal simulations), scikit-multiflow (stream scenarios), custom Monte Carlo sims, PyRIT (Red Teaming AI)
Differential privacy	Differentially private stochastic gradient descent, noise addition, private queries	Opacus (PyTorch), TensorFlow Privacy, SmartNoise (OpenDP)
Secure multiparty computation	Training without data centralization	CrypTen (Meta), PySyft (OpenMined), TF Encrypted, MOTION2NX
Federated and split learning	Distributed learning across sites	Flower, TensorFlow Federated, FedML, OpenFL (Intel)
Homomorphic encryption	Encrypted computation	HElib, PALISADE, SEAL (Microsoft), Concrete (Zama)
Data sovereignty/ governance	Compliance with data residency laws	Gaia-X connectors, lakeFS with region-based storage policies, DataHub lineage + policy enforcement

## Tip 7: Leverage the domain knowledge

In biomedical ML, integrating domain knowledge is essential for trustworthiness [17]. Biomedical expertise shapes datasets, model design, and interpretation, provides insights that align ML with biomedical logic, and grounds models in scientific reasoning.

Domain knowledge can be integrated at three key stages: pre-processing, in-processing, and post-processing [17].

In pre-processing, biological priors guide feature selection, engineering, and cleaning. For example, biomarkers or gene pathways help prioritize variables in genomics, while domain expertise informs experiments that capture disease heterogeneity and patient subpopulations [68].

In-processing embeds constraints directly into the learning objective [17]. This includes regularization enforcing monotonicity or conservation laws, and hybrid models that combine empirical data with physics-based simulators. Such strategies improve generalization and reduce biologically implausible results under distribution shifts [69].

Post-processing ensures outputs comply with clinical rules or logical constraints, such as disease taxonomies [70]. This step makes predictions more actionable and reliable. For example, one could exploit KEGG pathways in genomics [71] to validate prediction of biomedical annotations made on the Gene Ontology database [72]. Table 3 presents typical scopes along with the associated tasks and libraries to leverage domain knowledge.

**Table 3:** Typical scopes along with the associated tasks and libraries to leverage domain knowledge.

Scope	Tasks	Libraries
Pre-processing (domain-guided)	Feature selection, biological priors, biomarkers, cleaning, stratification	BioPython, Scanpy (single-cell), Bioconductor (R), scikit-learn feature selection, SHAP (feature importance), Omics-specific DBs (KEGG, Reactome, Gene Ontology), pandas-profiling/ ydata-profiling, Great Expectations
Experiment design/ cohort definition	Stratifying by disease subpopulations, handling heterogeneity	EHR data frameworks: FHIR APIs, OHDSI/OMOP CDM, pyomop, i2b2, DataSHIELD (privacy-preserving cohorting)
In-processing (knowledge-embedded)	Embedding priors/constraints in the model, hybrid physics-ML models	PyTorch constraints/ monotonic networks, TensorFlow Lattice, scikit-monotonic, DeepXDE (physics-informed), SimulAI/ SciML (Julia), PINNs frameworks, GPyTorch with structured kernels
Hybrid biomedical + ML models	Coupling simulators with ML	COPASI (biochem simulation), PySB (systems biology), NEURON (neuroscience), CellML/OpenCOR
Regularization with priors	Encourage plausible behavior under shifts	PyTorch custom regularizers, Keras custom loss layers, GPflow (structured priors)
Post-processing (knowledge-based validation)	Constraining outputs, aligning with biomedical logic, ontologies	OntoBio, PyOBO, OWLready2 (ontologies), SNOMED CT/ UMLS APIs, BioPortal, GOATOOLS
Rule-based combination with ML outputs	Logical/clinical constraints after model prediction	Drools (rule engine), PyKnow, Experta, Logica (Google)
Explainability aligned with biomedical knowledge	Making sure explanations match biological mechanisms	BioSHAP, Captum (with pathway grouping), Pathway2Vec, Knowledge Graph Embeddings (DGL-KE, PyKEEN)

## Tip 8: Follow open science best practices, and document everything

Trustworthy ML requires open technologies and data, making adherence to open science best practices essential. We recommend using open-source programming languages, openly sharing data (with proper authorization), releasing all code publicly, and publishing in open-access journals. Open-source tools such as Python or R enable free and reproducible experimentation, while proprietary software restricts access to those with licenses [73]. Where informed consent and ethics approval allow, patient data should be released through repositories like Zenodo, Figshare, Gene Expression Omnibus, or ArrayExpress. Both raw and processed data should be shared to maximize reuse and accelerate discovery [74]. Reproducibility further requires making all analysis code freely available on platforms such as GitHub or GitLab [75]. Open access publishing ensures that results are freely available worldwide, increases visibility in under-resourced regions, and is associated with higher citation rates [76].

Finally, we stress the importance of clear documentation, including well-annotated code [77] and a scientific diary tracking study progress and key methodological decisions [78]. Documenting choices such as evaluation metrics enhances transparency and strengthens trustworthiness. Of course, we know that sometimes it is impossible to share data in hospitals and biomedical research centers for privacy reasons, and we agree that patients’ rights should always be protected [48].

## Tip 9: Make your whole machine learning pipeline trustworthy

**Fig 1:**
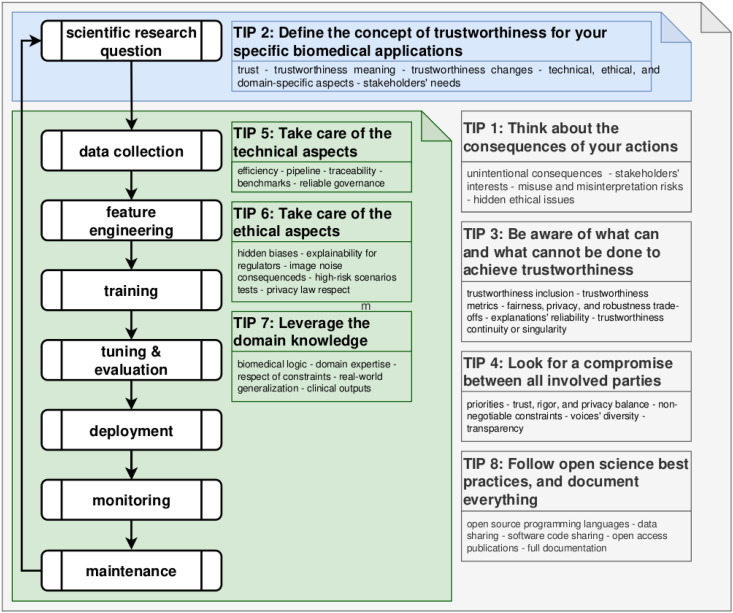
Representation of our tips for a trustworthy machine learning pipeline cycle in the biomedical sciences.

In this final tip, we argue that trustworthy biomedical ML requires rethinking the entire pipeline [79], from research question formulation to data collection, feature engineering, model training, evaluation, deployment, monitoring, and maintenance [16,41,44,80,81] (Fig 1).

While all stages benefit from the proposed tips, some have particular relevance in specific contexts. Tip 1 stresses the need to anticipate long-term consequences of design choices. Tip 3 highlights data and model limitations when defining system capabilities. Tip 4 calls for stakeholder involvement—including patients, clinicians, data scientists, and regulators—to ensure diverse perspectives. Tip 8 promotes open science practices, emphasizing transparency, documentation, and reproducibility. At the problem-definition stage, Tip 2 is crucial: it urges practitioners to define what trustworthiness means in context, aligning goals with ethical, clinical, and societal expectations.

During data collection and feature engineering, Tip 7 underscores the integration of domain expertise to embed relevant prior knowledge and ensure meaningful features. In model training, deployment, and maintenance, Tips 5, 6, and 7 collectively address technical, ethical, and domain-specific requirements, guiding the design of systems that are not only performant but also trustworthy. Trustworthiness is therefore not an afterthought, but a principle to be embedded at every stage of the pipeline [16,41,44,80,81].

A detailed workflow explaining the differences between a trustworthy machine learning pipeline and a traditional pipeline can be found in the study by Rasheed and coauthors [82]. An illustrative example of a wise usage of our eight tips is the study by Gardiner and colleagues [83]: they applied a transparent, trustworthy machine learning approach to genomics data and chemical structure information to predict kidney dysfunction, as a proxy for drug-induced renal toxicity, in rats.

## Conclusions

With the rapid growth of ML in biomedical sciences, there is increasing recognition of the need for trustworthy approaches among researchers, professionals, and regulators. Black-box effectiveness is no longer sufficient; evaluation must also consider technical, ethical, and domain-specific criteria. Yet, despite widespread efforts, few studies fully achieve trustworthiness.

In response, we present nine practical recommendations to strengthen ML in biomedical sciences. These tips, if adopted, can enhance the trustworthiness of any study involving ML, and are equally relevant to other scientific fields where ML plays a central role.
